# Towards Engineering an Orthogonal Protein Translation Initiation System

**DOI:** 10.3389/fchem.2021.772648

**Published:** 2021-10-26

**Authors:** Byeong Sung Lee, Woon Jong Choi, Sang Woo Lee, Byoung Joon Ko, Tae Hyeon Yoo

**Affiliations:** ^1^ Department of Molecular Science and Technology, Ajou University, Suwon, South Korea; ^2^ School of Biopharmaceutical and Medical Sciences, Sungshin Women’s University, Seoul, South Korea; ^3^ Department of Applied Chemistry and Biological Engineering, Ajou University, Suwon, South Korea

**Keywords:** translation initiation, non-canonical amino acid, initiator tRNA, amber codon, Methanococcus jannaschii tRNA

## Abstract

In the last two decades, methods to incorporate non-canonical amino acids (ncAAs) into specific positions of a protein have advanced significantly; these methods have become general tools for engineering proteins. However, almost all these methods depend on the translation elongation process, and strategies leveraging the initiation process have rarely been reported. The incorporation of a ncAA specifically at the translation initiation site enables the installation of reactive groups for modification at the N-termini of proteins, which are attractive positions for introducing abiological groups with minimal structural perturbations. In this study, we attempted to engineer an orthogonal protein translation initiation system. Introduction of the identity elements of *Escherichia coli* initiator tRNA converted an engineered *Methanococcus jannaschii* tRNA^Tyr^ into an initiator tRNA. The engineered tRNA enabled the site-specific incorporation of O-propargyl-l-tyrosine (OpgY) into the amber (TAG) codon at the translation initiation position but was inactive toward the elongational TAG codon. Misincorporation of Gln was detected, and the engineered system was demonstrated only with OpgY. We expect further engineering of the initiator tRNA for improved activity and specificity to generate an orthogonal translation initiation system.

## Introduction

Site-specific modification of the N-terminus of a protein is an attractive strategy to introduce unnatural groups with minimal effects on original protein functions. Protein termini tend to be exposed and flexible but not buried in the core (Christopher and Baldwin 1996; Jacob and Unger 2007). The N-terminal α-amino group is a good nucleophile, and its reactivity has been utilized to modify the N-terminus of proteins specifically. While the pKa of a lysine side-chain is around 10.5, the pKa values of the N-terminal amines have been reported between 6.8 and 9.1, with an average of 7.7 ± 0.5 (Serada and Mant, 1993). The reactivities of N-terminal and lysine side chain amines can be different at a given pH, and several chemical reactions have been reported as methods to modify the N-terminal amine groups specifically (Rosen and Francis 2017). Enzymes have been found to catalyze the peptide-bond formation reaction, and some of them have been used to modify proteins at their N-termini, including sortase (Mao et al., 2004, Proft 2010), intein (Evans and Xu 1999; Xu and Evans 2001), and proteases (Liebscher et al., 2014; Weeks and Wells 2020).

Biological methods for incorporating non-canonical amino acids (ncAAs) into proteins have become invaluable tools for protein engineering (Wang and Schultz 2004). In particular, ncAAs with reactivities can provide sites for protein modification (Wang et al., 2009; Wals and Ovaa 2014; Chin 2017). *Escherichia coli* wild-type and variant methionyl-tRNA synthetase (MetRS) can charge the *E. coli* initiator methionyl-tRNA (*Ec*-tRNA^fMet^) with isostructural analogs of methionine (Met), and several analogs with orthogonal reactivities have been successfully installed at the N-termini of proteins (Link et al., 2003; Johnson et al., 2010; Agostini et al., 2017; Tharp et al., 2020; Pagar et al., 2021). However, they were also incorporated into the internal Met positions, and thus, site-specific modification at the N-terminal position was not achieved, besides cases where there was no internal Met residue. Interestingly, *Ec*-tRNA^fMet^ charged with ncAA is translationally active for protein synthesis initiation in eukaryotes (Ngo et al., 2013). The system was demonstrated to tag the proteome N-termini but was unsuitable for producing homogeneous recombinant proteins because either ncAA or Met occupied the N-terminal positions. Budisa et al. proposed a clever strategy to incorporate azidohomoalanine at the protein synthesis initiation position by deleting the elongator tRNA^Met^ genes from the *E. coli* genome and introducing an orthogonal pair of MetRS/tRNA^Met^ from *Sulfolobus acidocaldarius* (Simone, Acevedo-Rocha et al., 2016). However, the system was partially successful because of the incomplete orthogonality of the system.

Orthogonal pairs of aminoacyl-tRNA synthetase (aaRS) and tRNA have been developed to introduce diverse ncAAs into proteins in a site-specific manner in various organisms (Wang and Wang 2012; Xiao et al., 2013; Smolskaya and Andreev 2019). These methods were designed based on codons that are not generally used for coding amino acids (such as stop codons (Liu and Schultz 2010) and four-base codons (Hohsaka et al., 2001; Anderson et al., 2004; Wang et al., 2012; Lee et al., 2017)). Strategies have also been reported to incorporate ncAAs into sense codons by modifying orthogonal pairs (Lee et al., 2015; Mukai et al., 2015). However, all orthogonal tRNAs are elongator tRNAs, and thus, cannot be used to install ncAAs at the N-termini of proteins. Recently, Söll et al. reported an engineered *Ec*-tRNA^fMet^ that enabled the initiation of protein synthesis with ncAAs (Tharp et al., 2020). The initiator tRNA, named itRNATy2, had an identity element in *Methanococcus jannaschii* tRNA^Tyr^ (*Mj*-tRNA^Tyr^) by mutations of A72G and the anticodon for the amber codon (CAU→CUA). The initiator tRNA was a substrate for *M. jannaschii* tyrosyl-tRNA synthetase (*Mj*-TyrRS), and the tRNAs charged with ncAAs could be used for *E. coli* protein synthesis initiation. Despite this success, the initiator tRNA had the C1:G72 base pair and could not distinguish between the initiation and elongation amber codons.

**FIGURE 1:**
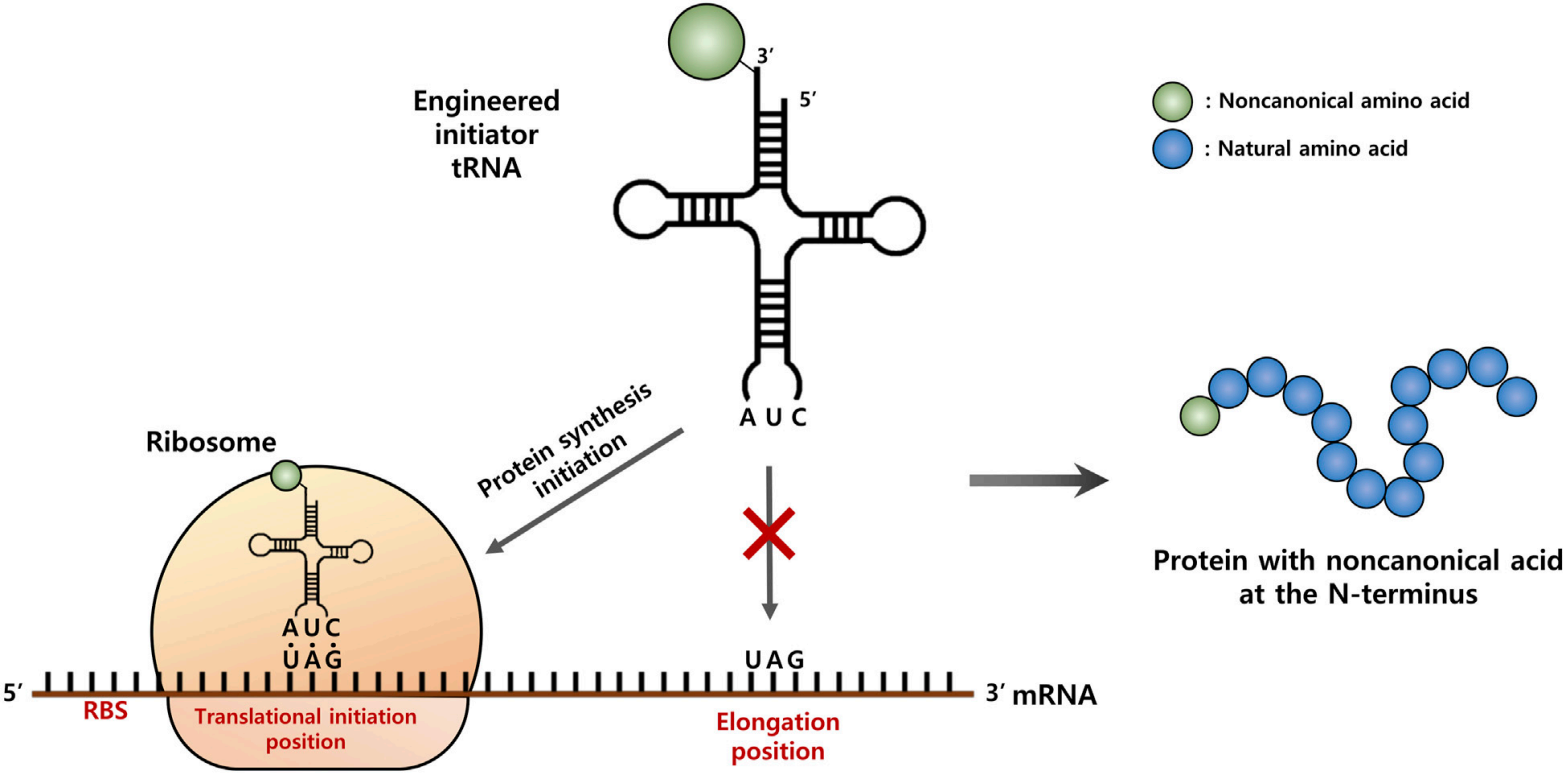
Schematic representation of the strategy for site-specific incorporation of a non-canonical amino acid (ncAA) into the start position of protein translation. An engineered initiator, tRNA_CUA_ charged with a ncAA is active toward the protein translation initiation with the amber (TAG) codon, but inactive for the elongation process.

It has been shown that the introduction of the main determinants of *Ec*-tRNA^fMet^ into elongator tRNAs, such as glutaminyl-tRNA and methionyl-tRNA, could convert them into tRNAs, enabling initiation of protein synthesis in *E. coli* (Varshney and RajBhandary 1990; Lee et al., 1991; Varshney et al., 1993). Based on the achievements of these studies, we aimed to engineer *Mj*-tRNA^Tyr^ into an initiator tRNA, which was intended to work only for an amber codon at the translation initiation position, but not for an internal amber codon (Figure 1). Identity elements for interactions with factors involved in the initiation of protein syntheses, such as methionyl-tRNA transformylase (MTF), the P-site of the 30S ribosomal subunit, and initiation factor-2 (IF-2), were introduced into *Mj*-tRNA^Tyr^. The engineered *Mj*-tRNA was active toward the initiation amber codon, but did not support the coding of internal amber codons. We believe that the results obtained in this study open a new route for developing an orthogonal translation initiation system to incorporate ncAAs at the N-termini of proteins.

## Materials and Methods

### Plasmid Construction

The plasmids, primers, and proteins used in this study are shown in Table 1; Supplementary Tables S1, S2, respectively. The synthesized initiator tRNA genes, including the ProK promoter and terminator (*Mj*-itRNA-1 and *Mj*-itRNA-2; the DNA sequences are shown in Supplementary Table S3) were cloned into the modified pEVOL (Young et al., 2010) plasmid, in which one copy of the azidophenylalaninyl-tRNA synthetase gene (AzF-RS) is located under the AraBAD promoter (Lee et al., 2015), using the *ApaL*I and *Xho*I sites. The resulting plasmids were named pSEPL773 for *Mj*-itRNA-1 and pSPEL541 for *Mj*-itRNA-2. The methionyl-tRNA transformylase (MTF) gene was amplified (primers 1 and 6) from the chromosome of *E. coli* DH10β and then cloned into the plasmid containing itRNA-1 (pSPEL773) or itRNA-2 (pSPEL541) using the *Nde*I and *Pst*I sites. The two *Pst*I sites present in the MTF gene were removed by assembly PCR using primer pairs (primers 2 and 3 for the first *Pst*I site; primers 4 and 5 for the second *PstI* site). The MTF gene was located under the glnS promoter. The resulting plasmids were named pSPEL527 (MTF/*Mj*-itRNA-1) and pSPEL528 (MTF/*Mj*-itRNA-2), respectively. The initiator factor-2 (IF-2) gene was amplified (primers 7 and 14) from the chromosome of *E. coli* DH10β and then cloned into the plasmid containing itRNA-1 (pSPEL773) or itRNA-2 (pSPEL541) using the *Nde*I and *Pst*I sites; the three *Pst*I sites present in the IF-2 gene were removed by assembly PCR using two pairs of primers (primers 8 and 9 for the first *Pst*I site; primer 10 and 11 for the second *Pst*I site; primers 12 and 13 for the third *Pst*I site). The resulting plasmids were named pSPEL780 (IF-2/*Mj*-itRNA-1) and pSPEL562 (IF-2/*Mj*-itRNA-2), respectively. Plasmids expressing both MTF and IF-2 were constructed by cloning the IF-2 gene with a ribosome-binding sequence (Kinneer et al., 2019) into the *Pst*I site of pSPEL527 or pSPEL528, and the orientation of the RBS-IF-2 genes was confirmed by DNA sequencing. The resulting plasmids were named pSPEL781 (MTF/IF-2/*Mj*-itRNA-1) and pSPEL563 (MTF/IF-2/*Mj*-itRNA-2), respectively.

**TABLE 1:** Plasmids used in this study.

Name	Characteristics^a^	Source
pEVOL	*Mj*-tRNA^Tyr^, AzFRS, Cm^R^, p15A ori	Young et al. (2010)
pSPEL773	pEVOL-*Mj*-itRNA-1, AzFRS, Cm^R^, p15A ori	This study
pSPEL541	pEVOL-*Mj*-itRNA-2, AzFRS, Cm^R^, p15A ori	This study
pSPEL527	pEVOL-*Mj*-itRNA-1, AzFRS, MTF, Cm^R^, p15A ori	This study
pSPEL528	pEVOL-*Mj*-itRNA-2, AzFRS, MTF, Cm^R^, p15A ori	This study
pSPEL780	pEVOL-*Mj*-itRNA-1, AzFRS, IF-2, Cm^R^, p15A ori	This study
pSPEL562	pEVOL-*Mj*-itRNA-2, AzFRS, IF-2, Cm^R^, p15A ori	This study
pSPEL781	pEVOL-*Mj*-itRNA-1, AzFRS, MTF, IF-2, Cm^R^, p15A ori	This study
pSPEL563	pEVOL-*Mj*-itRNA-2, AzFRS, MTF, IF-2, Cm^R^, p15A ori	This study
pQE-80L	Expression vector, Amp^R^, ColE1 ori	Qiagen
pSPEL542	pQE-80L-TAG-Z domain, Amp^R^, ColE1 ori	This study
pBbE6a	Expression vector, Amp^R^, ColE1 ori	Lee et al. (2011)
pSPEL530	pBbE6a-TAG-11.3.3, ColE1 ori	This study
pGEX-4T-1	Expression vector with GST, Amp^R^, pBR322 ori	Amersham Bioscience
pSPEL236	pGEX-4T-1-GST-TAG-Z domain, Amp^R^, pBR322 ori	This study
pBbS2K-ProRS	pBbS2K-Prolyl tRNA synthetase, Kan^R^, SC101 ori	Lee et al. (2015)

a Mj, Methanococcus jannaschii; AzFRS, azidophenylalanyl tRNA synthetase; Cm, chloramphenicol; Amp, ampicillin; Kan, kanamycin; MTF, methionyl-tRNA transformylase; IF-2, initiation factor-2.

A DNA double helix for a multiple cloning site (*Nco*I-*BamH*I-*Hind*III-*Not*I-*Xho*I-His_6_ Tag) prepared by annealing primers 15 and 16 was phosphorylated using T4 polynucleotide kinase and then ligated into the pBbE6a (Lee et al., 2011) plasmid digested with *Nde*I and *Xho*I. The RBS-GFP gene with the TAG codon at the translation initiation site was generated by PCR amplification using the 11.3.3 GFP gene (Yoo et al., 2007) as a template with primers 17 and 18, and the product was cloned into the modified pBbE6a mentioned above using the *EcoR*I and *Hind*III sites, resulting in pSPEL530. The synthesized gene of the Z domain (the amino acid sequence in Supplementary Table S2) with an amber (TAG) codon at the translation initiation site was cloned into pQE-80L (Qiagen) using the *EcoR*I and *Hind*III sites, resulting in pSEPL542. The Z domain gene was amplified using pSPEL542 as a template with primers 19 and 20; the product was cloned into pGEX-4T-1 (Amersham Bioscience) *EcoR*I and *Xho*I sites. To introduce an amber (TAG) codon between the glutathione-S-transferase (GST) and Z domain, an annealed DNA double helix of primers 21 and 22 was phosphorylated using T4 polynucleotide kinase and then cloned into the pGEX-4T-1 with the Z domain gene using the *BamH*I and *EcoR*I sites, resulting in pSPEL236.

### Non-Canonical Amino Acid Incorporation

The plasmid coding Z domain (pSPEL542) or GFP (pSPEL530) with an amber (TAG) codon at the translation initiation position was co-transformed with one of the pEVOL derivatives (pSPEL731, 541, 527, 528, 780, or 562) and pBbS2K-ProRS (Lee et al., 2015) encoding *E. coli* prolyl-tRNA synthetase under the tetracycline-inducible promoter into *E. coli* DH10β. The cells were grown in 2xYT media containing 200 μg/ml ampicillin, 34 μg/ml chloramphenicol, and 35 μg/ml kanamycin at 37°C until the OD_600_ reached 0.5. To induce AzF-RS and prolyl-tRNA synthetase expression, 0.2% L-arabinose and 50 nM anhydrotetracycline were added to the culture. When OD_600_ reached 1.0, 1 mM isopropyl β-D-thiogalatoside and 1 mM ncAA were added to express the model protein. After 3 h, the cells were harvested by centrifugation at 14,000 × g at 4 C for 20 min, and the cell pellets were stored at −20°C until use.

### Copper (I)-Catalyzed Click Reaction and Western Blotting

The cell pellets were resuspended in 1% SDS solution and heated at 95°C for 5 min for lysis. The supernatant obtained after centrifugation at 14,000 × g for 5 min was subjected to a copper (I)-catalyzed click reaction with biotin-PEG_3_-azide (Click Chemistry Tools) following the method described (Hong et al., 2009). The reaction mixtures were analyzed by western blotting using a streptavidin-horseradish peroxidase (HRP) conjugate (Click Chemistry Tools) and an anti-His_5_-HRP conjugate (Sigma-Aldrich, St. Louis, MO).

### Protein Purification

The Z-domain protein with an N-terminal His6 tag was purified using a Ni-immobilized resin (Clontech, Mountain View, CA) under native conditions, following the manufacturer’s instructions. The cell pellets stored at −20 C were resuspended in lysis buffer (50 mM sodium phosphate, 300 mM sodium chloride, 20 mM imidazole, pH 7.4) and incubated on ice for 30 min with 50 μg/ml lysozyme. After sonication, the lysed cells were centrifuged at 9,300 × g at 4°C for 1 h. The supernatant was incubated at 4°C for 1 h with Ni resin, pre-equilibrated with lysis buffer. The resin was loaded onto a gravity flow column (Thermo Fisher Scientific, Waltham, MA) and washed three times with washing buffer (50 mM sodium phosphate, 300 mM sodium chloride, 40 mM imidazole, pH 7.4). The protein was eluted using elution buffer (50 mM sodium phosphate, 300 mM sodium chloride, 300 mM imidazole, pH 7.4). The protein solutions were buffer-exchanged with phosphate buffer saline (PBS) solution (10 mM KH2PO4, 150 mM NaCl, pH 7.4) using a centrifugal filter unit (Millipore, 3000 MWCO).

### Mass Spectrometry

The intact masses of the proteins were analyzed using a Waters ACQUITY I class UPLC system (Milford, MA) with an ACQUITY UPLC Protein BEH C4 column (2.1 mm × 100 mm, 1.7 μm particle size; Waters). The mobile phases were 0.1% formic acid in water (eluent A) and 0.1% formic acid in acetonitrile (eluent B). The gradient applied was: 0–3 min, 5% eluent B; 3–13 min, linear increase to 50% eluent B at 0.2 ml/min. The eluent was injected into a Thermo Orbitrap Elite (Thermo Fisher Scientific, Waltham, MA) and ionized with an electrospray source. MS spectra were acquired in the mass range of 400–2,000 m/z and 120,000 resolution at m/z 200. The deconvoluted mass spectra were generated using Protein Deconvolution 2.0 (Thermo Fisher Scientific, Waltham, MA).

### N-Terminal Sequencing

The purified Z-domain protein was transferred to a PVDF membrane (0.45 μm pore size; Pall), and the membrane was washed with ultrapure water and dried in air. The membrane was subjected to an N-terminal sequencing analysis using Procise® LC492 Protein Sequencing System (Applied Biosystems, Waltham, MA). The retention times of phenylthiohydantoin (PTH)-amino acids were compared with those of the standards (Tokyo Chemical Industry, Tokyo). PHT-OpgY was synthesized following a previously published method (Steiman et al., 1985).

## Results

Introduction of the identity elements of Ec-tRNA^fMet^ converted Mj-tRNA^Tyr^ into an initiator tRNA

**FIGURE 2:**
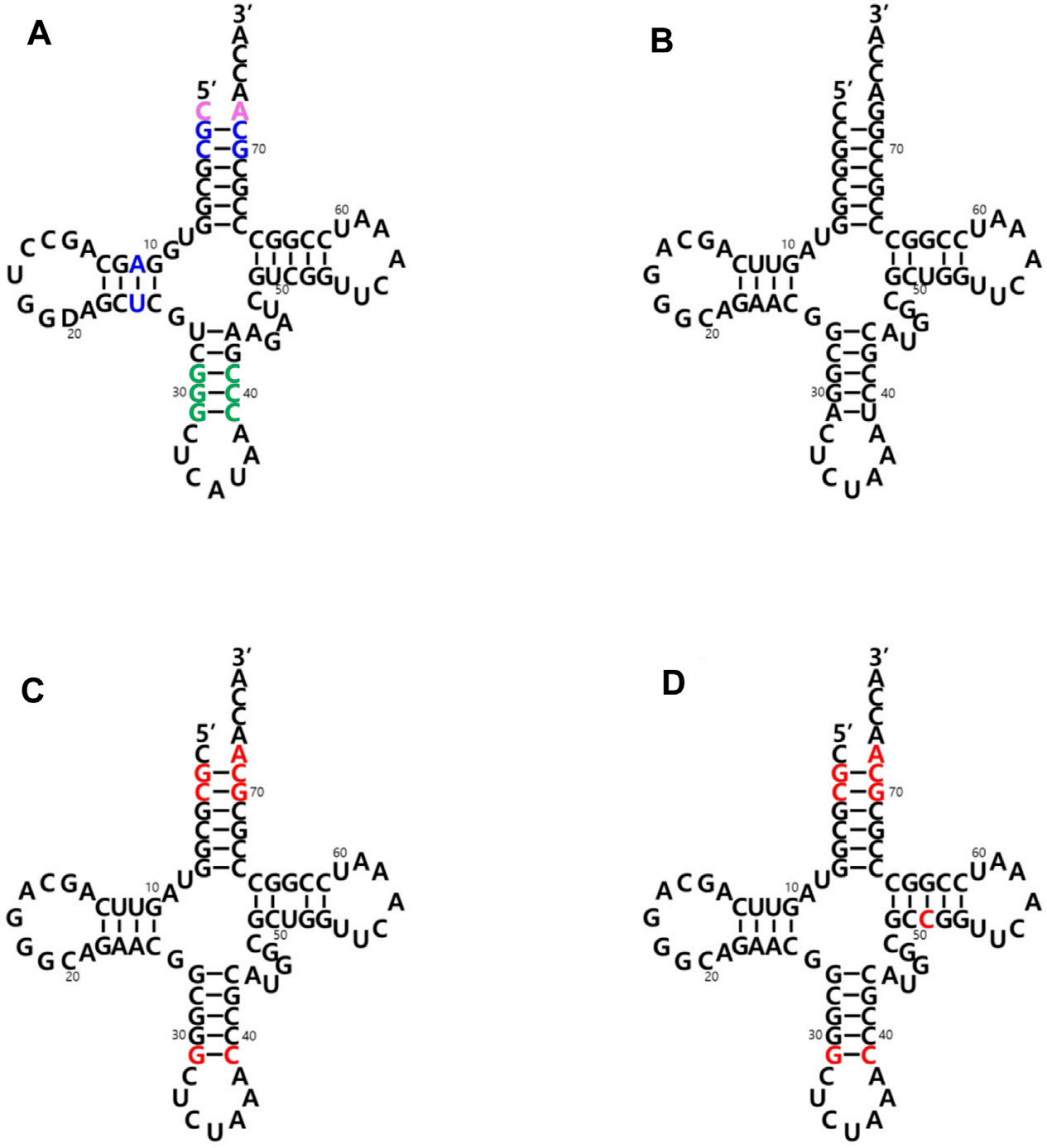
Cloverleaf depiction of tRNAs used in this study. **(A)** *E. coli* initiator tRNA (Ec-tRNA^fMet^). The two main identity elements of *Ec*-tRNA^fMet^ are the absence of a Watson-Crick base pair between positions C1 and A72 in the acceptor stem (in pink) and three consecutive G:C base pairs in the anticodon stem (in green). Some additional elements playing a role in the formylation reaction are shown in blue. **(B)** Engineered *M. jannaschii* tyrosyl-tRNA_CUA_ (*Mj*-tRNA^Tyr^). **(C)** *Mj* initiator tRNA_CUA_-1 (*Mj*-itRNA-1). **(D)** Mj initiator tRNA_CUA_-2 (*Mj*-itRNA-2). The changed nucleotides in *Mj*-itRNA-1 and *Mj*-itRNA-2 compared with *Mj*-tRNA^Tyr^ are marked by red letters.

The two main identity elements of the *E. coli* initiator tRNA (*Ec*-tRNA^fMet^) are 1) the absence of a Watson-Crick base pair between positions 1 and 72 in the acceptor stem (pink in Figure 2A) and 2) three consecutive G:C base pairs in the anticodon stem (green in Figure 2A). The C1:A72 mismatch plays a critical role in the interaction with MTF, and the 3G: C pairs are important for targeting the initiator tRNA to the P-site of the 30S ribosomal subunit (Kozak 1999; Laursen et al., 2005; Louise. et al., 2009). It was previously demonstrated that an *E. coli* glutaminyl-tRNA (*Ec*-tRNA^Gln^) could be converted into an initiator tRNA by introducing these two determinants. The engineered tRNA with the CUA anticodon for the amber (TAG) nonsense codon was activated by the endogenous glutaminyl-tRNA synthetase (GlnRS), and the initiator tRNA charged with Gln enabled the synthesis of recombinant proteins with a TAG codon at their initial position (Varshney et al., 1993). Based on these results, we introduced the two elements into an engineered *Mj*-tRNA^Tyr^ (Wang and Schultz 2001) (Figure 2B) by mutations of G72→A72 for the C1:A72 mismatch and A31:U39→G31:C39 for the three consecutive G:C pairs in the anticodon stem. In addition to the C1:A72 mismatch, several other elements in the acceptor and the D stem (blue in Figure 2A) have been reported to play a role in the formylation by MTF (RajBhandary 1994, Louise, L. et al., 2009). Based on a previously published report in which *Mj*-tRNA^Tyr^ was engineered for its orthogonality to the 20 endogenous aminoacyl-tRNA synthetases of *E. coli* (Guo et al., 2009), several additional mutations were introduced into the acceptor stem. Since any changes in the A11:U24 pair significantly inhibited charging the tRNA in our previous experiments (unpublished results), we decided not to introduce the element in the D stem into *Mj*-tRNA^Tyr^, and the resulting molecule was named *Mj*-itRNA-1 (Figure 2C). The C51G52 motif was reported to interact with IF-2 (Louise, L. et al., 2009), although it is weak, and the element was additionally introduced into *Mj*-itRNA-1 by a mutation of U51→C51, resulting in *Mj*-itRNA-2 (Figure 2D).

**FIGURE 3:**
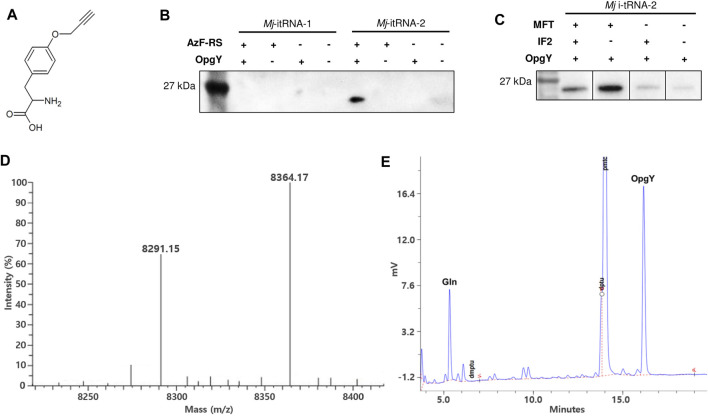
Incorporation of non-canonical amino acids into the translation initiation position. **(A)** O-propargyl-l-tyrosine (OpgY). **(B)** Comparison of *Mj*-itRNA-1 and *Mj*-itRNA-2 for the OpgY incorporation into the TAG codon located at the N-terminus of GFP. **(C)** Effects of additional expression of *E. coli* methionyl-tRNA transformylase (MTF) or *E. coli* initiation factor-2 (IF-2) on the protein synthesis initiation at the TAG codon. Each cell lysate was subjected to the Cu(I)-catalyzed click reaction with biotin-azide, and then the blot was probed with a streptavidin-horse radish peroxidase (HRP) conjugate **(B, C)**. The uncropped raw data were shown in Supplementary Figures S2B, S2C . **(D)** Deconvoluted electrospray ionization mass spectrum of the purified Z domain. The protein has the TAG codon at the translation initiation position. The calculated mass when OpgY is incorporated into the TAG codon is 8,364.06 Da, and that with Gln is 8,290.96 Da. **(E)** Edman sequencing result for the first residue of the purified Z domain expressed with OpgY. The results for the second to the fifth residue are shown in Supplementary Figure S4.

The start codon of a GFP gene (Yoo et al., 2007) was changed to the TAG amber codon, and the gene was cloned into pBbE6a (Lee et al., 2011). The tRNA gene in the pEVOL-AzF plasmid (Young et al., 2010) was replaced with *Mj*-itRNA-1 or *Mj*-itRNA-2. pEVOL-AzF encodes an *Mj*-TyrRS variant (AzF-RS) that is active toward both AzF and O-propargyl-l-tyrosine (OpgY) (Lee et al., 2015). The resulting plasmids were transformed into *E. coli* DH10β with pBbS2k-ProRS, which overexpresses *E. coli* prolyl-tRNA synthetase (*Ec*-ProRS) and suppresses the mischarging of *E. coli* prolyl-tRNA with ncAAs (Lee et al., 2015). Expression of AzF-RS and *Ec*-ProRS was induced by l-arabinose and anhydrotetracycline, respectively, and the GFP protein with the TAG codon at its N-terminus was expressed by isopropyl β-D-thiogalatopyranoside in the presence of OpgY (Figure 3A). The cell lysate reacted with biotin-azide via the Cu(I)-catalyzed click reaction, and the products were analyzed by western blotting using a HRP conjugate (Figure 3B). The signal indicates that the protein has an alkyne group of OpgY. Only when all *Mj*-itRNA-2, AzF-RS, and OpgY were present, the western signal was detected; which suggests that OpgY was incorporated into the TAG codon located at the initial position of GFP. That is, *Mj*-itRNA-2 was charged with OpgY by AzF-RS, and the α-amine group of OpgY in the aminoacylated tRNA was probably formylated by MTF, and then the resulting tRNA, formyl-OpgY-*Mj*-itRNA-2, supported the translation initiation with the amber codon.

### Overexpression of MTF Increased the Efficiency of the Protein Synthesis Initiation with ncAA

Even though *Mj*-itRNA-2 enabled the initiation of protein synthesis at the amber codon, its interactions with the factors involved in forming the 30S initiation complex might be relatively weak compared with those of *Ec*-tRNA^fMet^. An approach to address this would be to increase the concentrations of these factors instead of engineering the tRNA further. The three interactions of MTF, 30S ribosome, and IF-2 with *Mj*-itRNA-2 aminoacylated with OpgY could be considered to increase translation initiation with OpgY. We decided to explore the overexpression of MTF or IF-2; increasing the concentration of the 30S ribosome could affect various aspects of physiology and thus was not tested in this study. The genes for MTF or IF-2 were cloned under the constitutive Gln promoter of pEVOL-AzF; a biscistronic gene was used to express MTF and IF-2 simultaneously. Additional expression of MTF significantly improved the incorporation of OpgY into the amber codon at the initiation position of GFP, while the effect of overexpressing IF-2 was marginal (Figure 3C). This observation suggested that the formylation of the α-amine group of OpgY-*Mj*-itRNA-2 was the rate-determining step in translation initiation with the TAG codon. Expressing IF-2 in addition to MTF via the biscistronic construct decreased the signal compared with the case of expressing only MTF. This might be because the expression of IF-2 decreased the level of MTF due to the limited resources for transcription and translation in expressing multiple genes. The aminoacyl-tRNA synthetase of AzF-RS was originally engineered to activate AzF (Chin et al., 2002) and incorporate AzF into the initiation TAG codon was attempted using the same *E. coli* strain. However, unlike OpgY, *Mj*-itRNA-2 did not support protein translation initiation with AzF (data not shown). In the case of *Mj*-itRNA-1, additional expression of MTF or IF-2 did not result in detectable incorporation of OpgY into the initiation TAG codon (data not shown).

### The TAG Start Codon was Encoded with Either ncAA or Gln by Mj-itRNA-2

A small protein (Z domain (Nilsson et al., 1987)) was used to evaluate OpgY incorporation using a mass spectrometry. The start codon of Z domain was changed to TAG codon, and the resulting gene was cloned into pQE-80 L. The Z domain was expressed using *E. coli* cells expressing *Mj*-itRNA-2, AzF-RS, MTF, and ProRS in the presence of OpgY. The purified protein was analyzed by liquid chromatography-mass spectrometry. The calculated mass of the Z domain with OpgY at its N-terminus was 8,364.06 Da, and a mass of 8,364.17 was detected (Figure 3D). This result, along with the western blot results (Figures 3B,C), indicated that OpgY was incorporated into the TAG codon of the translation initiation position. However, another mass peak (8,291.15 Da) was observed in the Z-domain sample. Since the mass of the wild-type Z domain (Met at its N-terminus) is 8,294.03 Da (Supplementary Figure S3: the observed mass was 8,294.12), it was suspected that a canonical amino acid besides Met was incorporated into the TAG codon. The N-terminal residue of the Z domain was determined by the Edman degradation analysis, and two residues, OpgY and Gln, were detected (Figure 3E). The peaks for Gln and OpgY were integrated (597 for Gln and 1477 for OpgY), and the ratio of OpgY to Gln incorporated into the TAG position was calculated as 2.55. The mass of 8,291.15 Da was consistent with the calculated mass (8,290.96 Da) when Gln was incorporated into the TAG codon.

### Mj-itRNA-2 Did Not Support the Incorporation of ncAA into an Internal Amber Codon

**FIGURE 4:**
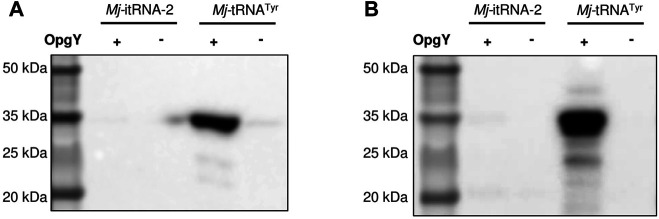
Suppression of an elongational TAG codon with *Mj*-itRNA-2. There is a TAG codon between GST and the Z domain-His_6_, and the fusion protein was expressed with *Mj*-itRNA-2 or *Mj*-tRNA^Tyr^. Each cell lysate was subjected to the Cu(I)-catalyzed click reaction with biotin-azide, and then the blot was probed with an anti-His_5_-HRP conjugate **(A)** or a streptavidin-HRP conjugate **(B)**. The uncropped raw data were shown in Supplementary Figures S5B, S5C.

Next, we examined whether *Mj*-itRNA-2 could function as an elongator tRNA. The two main elements of *Ec*-tRNA^fMet^, the C1:A72 mismatch, and the three consecutive G:C pairs in the anticodon stem, are known to distinguish the initiator tRNA from elongator tRNAs and to prevent it from being involved in the translation elongation process (Laursen et al., 2005). In addition to the two elements, *Mj*-itRNA-2 had mutations in the acceptor and the D stem; the T stem has also been reported to interact with *E. coli* elongation factor Tu (Guo et al., 2009). A plasmid was constructed by introducing the TAG codon between GST and the Z domain, and the protein had a C-terminal His_6_-tag. The GST-TAG-Z protein was expressed with either *Mj*-tRNA^Tyr^ (Figure 2B) or *Mj*-itRNA-2, and the cell lysates were analyzed by western blotting after the Cu(I)-catalyzed click reaction with biotin-azide. While OpgY was efficiently incorporated into the TAG codon with the elongator *Mj*-tRNA^Tyr^, *Mj*-itRNA-2 could not suppress the internal nonsense codon (Figure 4). These results indicate that *Mj*-itRNA-2 1) is charged with ncAA, 2) initiates protein translation at the TAG codon, and 3) is not active in the elongation process of protein synthesis. *Mj*-itRNA-2 is the first reported tRNA enabling the orthogonal incorporation of ncAA into the amber codon only at the translation initiation position.

## Discussion

Installation of ncAAs into specific positions of the protein has provided invaluable tools for not only studying proteins but also engineering them. Orthogonal pairs of tRNA/aaRS have been engineered for these purposes, and novel proteins with various unnatural functional groups have been created. These systems have been developed to target the elongation process of protein synthesis. However, a method to incorporate ncAAs at the translation initiation position in a site-specific manner has rarely been reported. This may be attributed to the difficulty in engineering functional initiator tRNAs compared with elongation tRNAs. The protein synthesis initiation process of *E. coli* is distinct from the elongation process, and the initiator tRNA (*Ec*-tRNA^fMet^) plays an important role in this process. *Ec*-tRNA^fMet^ interacts with MTF, 30S ribosome, and IF-2(RajBhandary 1994; Laursen et al., 2005); the identity elements for these interactions also prevent association with the elongation process. In this study, we introduced the identity elements of *Ec*-tRNA^fMet^ into an elongator *Mj*-tRNA^Tyr^ variant. One of the designed initiator tRNAs, *Mj*-itRNA-2, incorporated OpgY into the TAG codon at the translation initiation position, but was not active for an internal TAG codon.

Overexpression of MTF significantly improved the efficiency of translation initiation with OpgY, and the result implied that OpgY-*Mj*-itRNA-2 was not an efficient substrate for MTF. The same system failed to initiate protein translation with AzF, even though AzF-RS is also active toward ncAA. The identity of amino acids linked to an initiator tRNA affects the activity of MTF (Schmitt et al., 1998; Laursen et al., 2005). It is plausible that AzF-*Mj*-tRNA-2 was not formylated, considering the structural difference between OpgY and AzF. The elements present in the acceptor stem were introduced for interaction with MTF, but some other elements such as A11: U24 in the D stem (Lee et al., 1991; Ramesh et al., 1997) were not tested in this study because of the detrimental effects of mutations in this region in our previous experiments. In addition to OpgY, Gln was incorporated into the start TAG codon, which suggests that *Mj*-itRNA-2 was charged with Gln, probably by the endogenous glutaminyl-tRNA synthetase (*Ec*-GlnRS). These results suggest that further engineering of the initiator tRNA, particularly for enhancing the interaction with MTF and preventing the aminoacylation with Gln by Ec-GlnRS, could improve translation initiation efficiency with ncAAs. The newly engineered initiator tRNAs would enable developing an orthogonal translation initiation system active only for the TAG start codon. Furthermore, with many *Mj*-TyrRS variants engineered toward various ncAAs, a repertoire of functional groups would be available for site-specific incorporation at the N-termini of proteins.

## Data Availability

The original contributions presented in the study are included in the article/Supplementary Material, further inquiries can be directed to the corresponding author.
