# Supplementary material for: Towards Engineering an Orthogonal Protein Translation Initiation System
Source: Front Chem. 2021 Oct 26;9:772648. doi: 10.3389/fchem.2021.772648 (PMC8576571; doi:10.3389/fchem.2021.772648)
Supplement: Supplementary file 1 [file DataSheet1.docx]

Supplementary Material


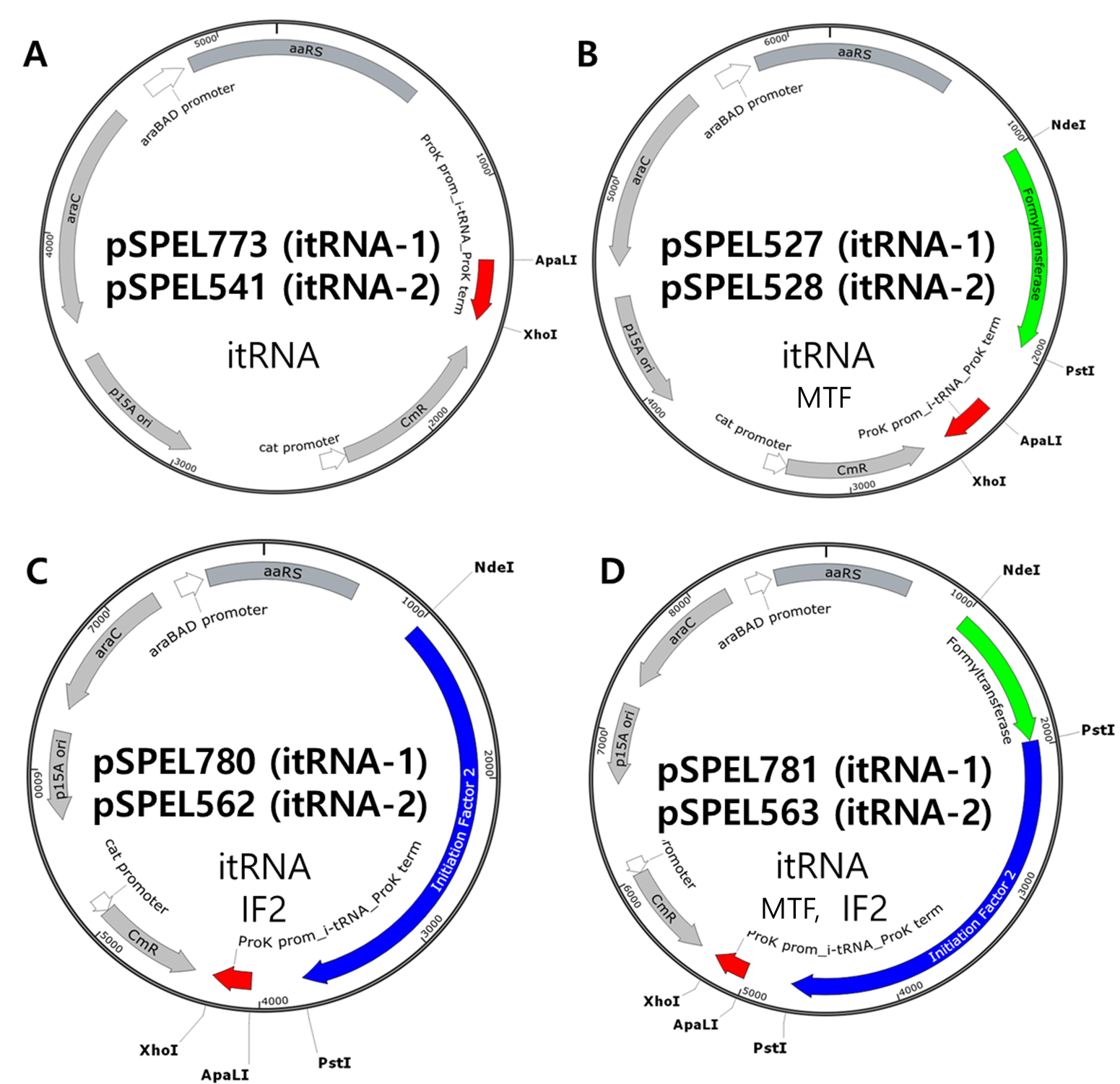


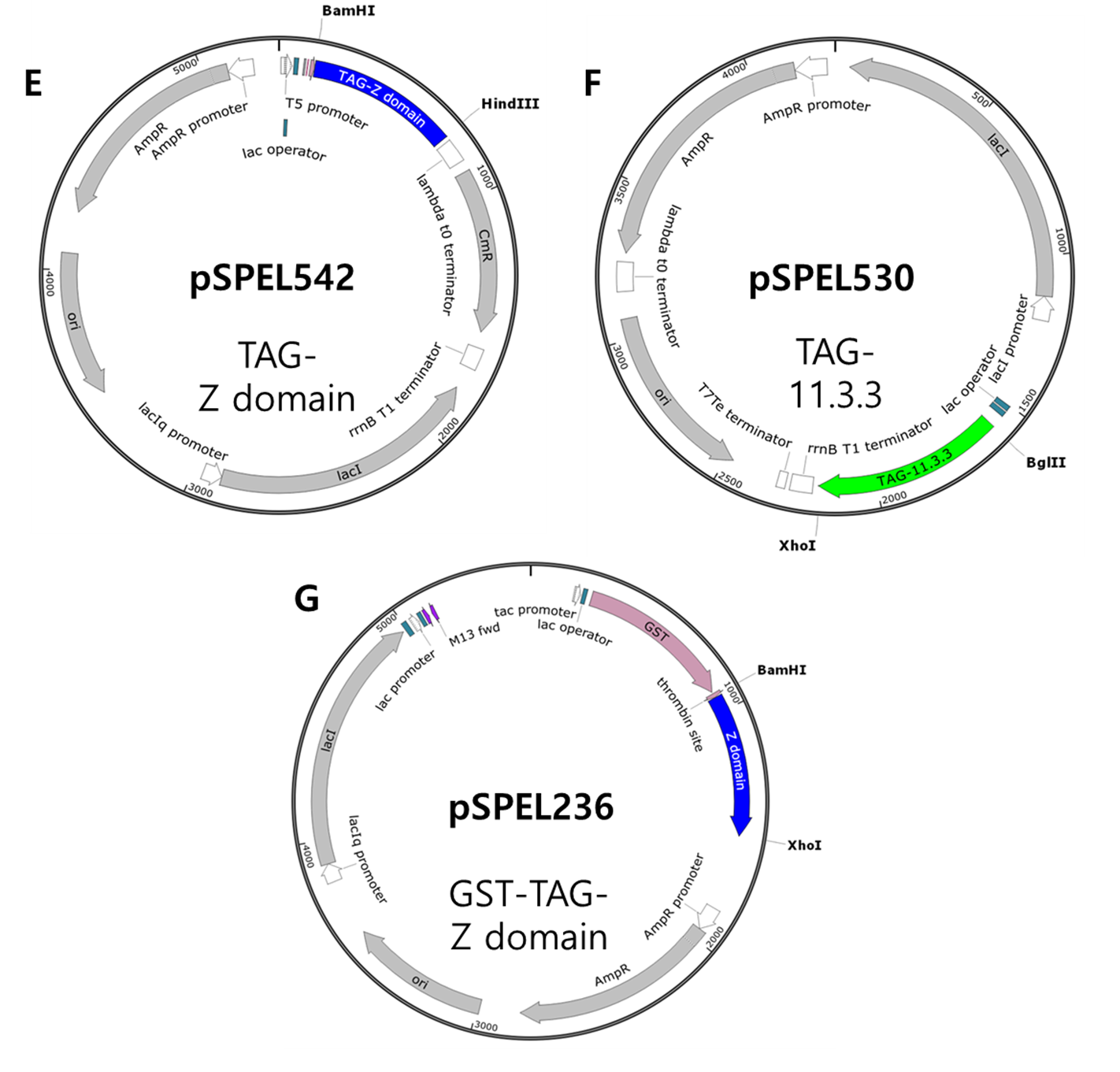


**Supplementary Figure S1.** Plasmid maps. **(A)** pEVOL derivative expressing itRNA, **(B)** pEVOL derivative expressing itRNA and FMT, **(C)** pEVOL derivative expressing itRNA and IF2, **(D)** pEVOL derivative expressing itRNA, MTF, and IF2, **(E)** pQE-80L derivative expressing the TAG-Z domain, **(F)** pBbE6a derivative expressing TAG-11.3.3, **(G)** pGEX-4T1 derivative expressing GST-TAG-Z domain.


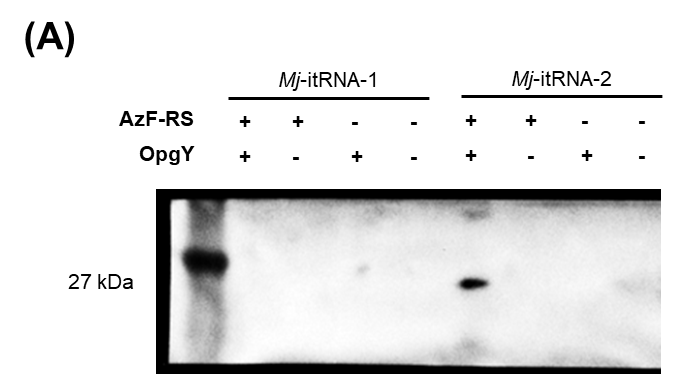


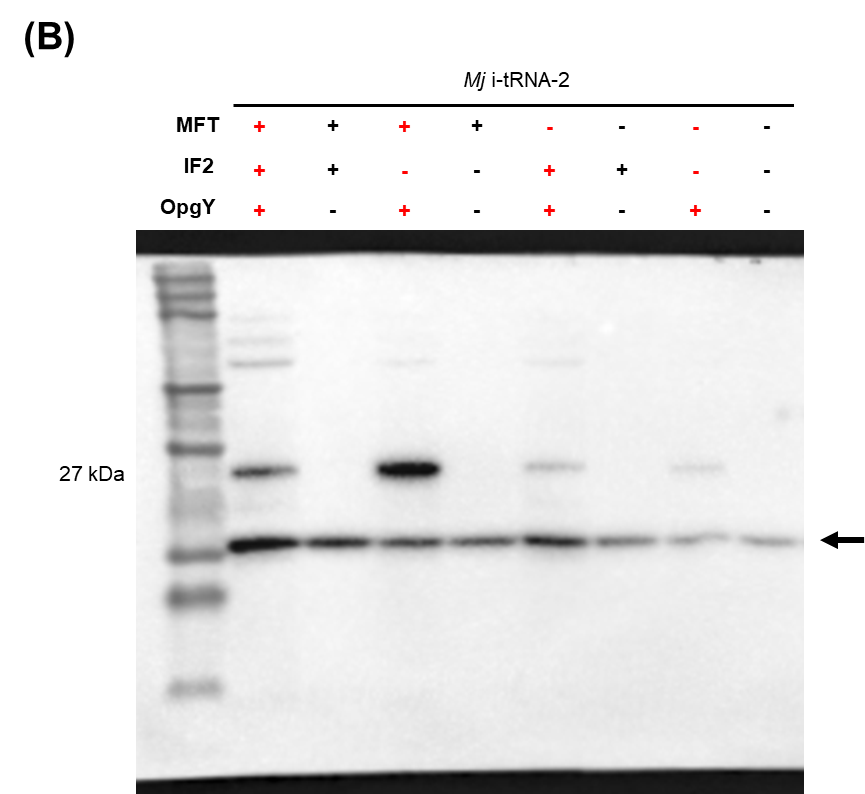


**Supplementary Figure S2.** **(A)** The unprocessed western blot data figure 3 panel B,
**(B)** The unprocessed western blot data figure 3 panel C. The samples in figure 3 panel C were shown in red. The bands indicated by the arrow are an endogeneous biotinated protein of *E. coli*, biotoin carboxy carrier protein.


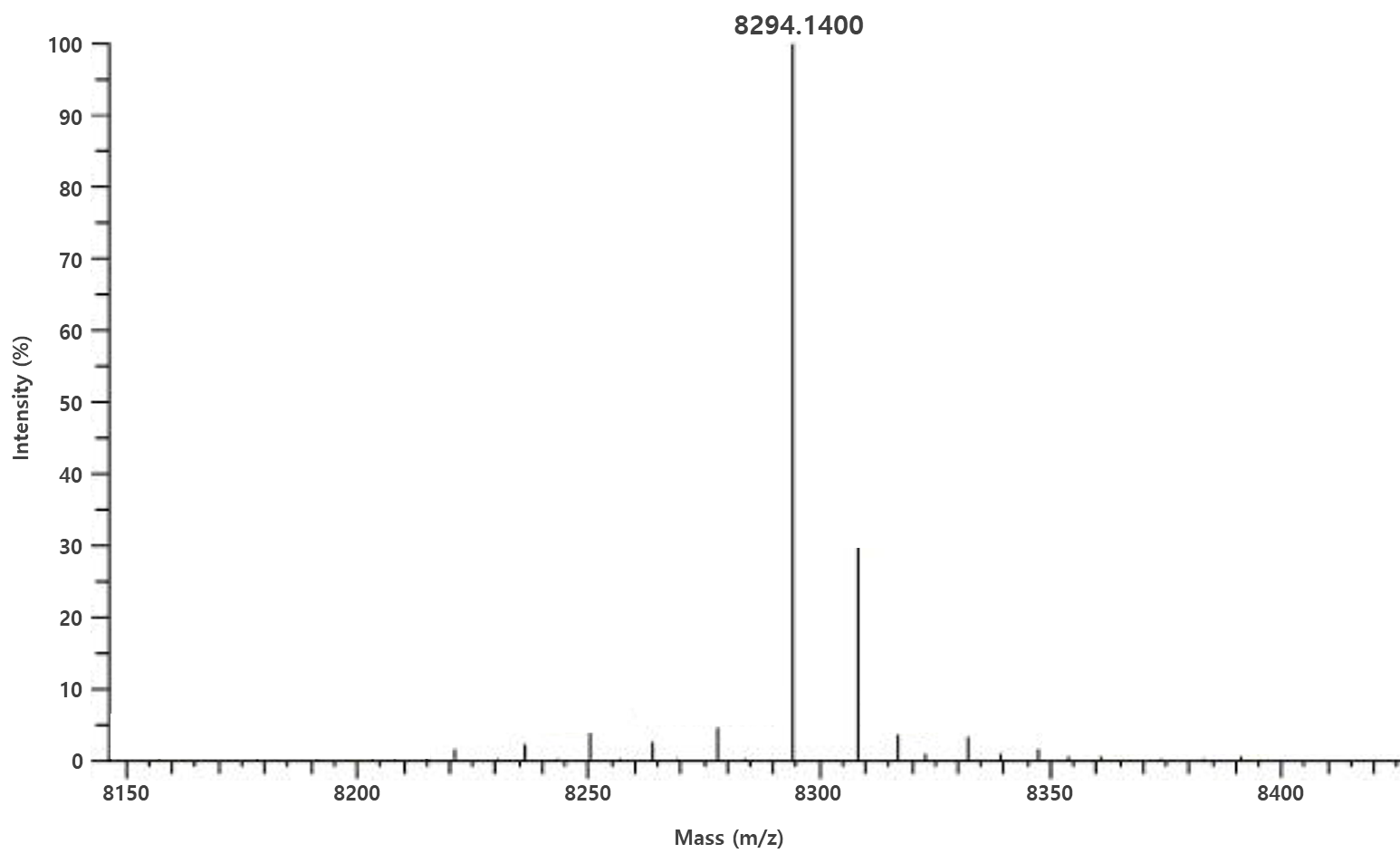


**Supplementary Figure S3.** Deconvoluted ESI-mass spectra of the wild-type Z domain. The mass for the wild-type Z domain, which has the N-terminal Met, was 8,294.14; the calculated mass is 8,294.03.


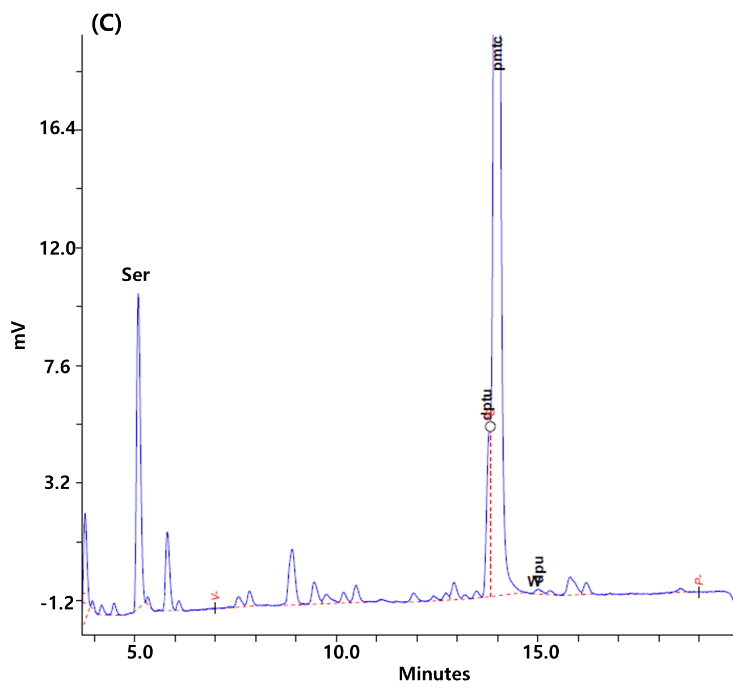

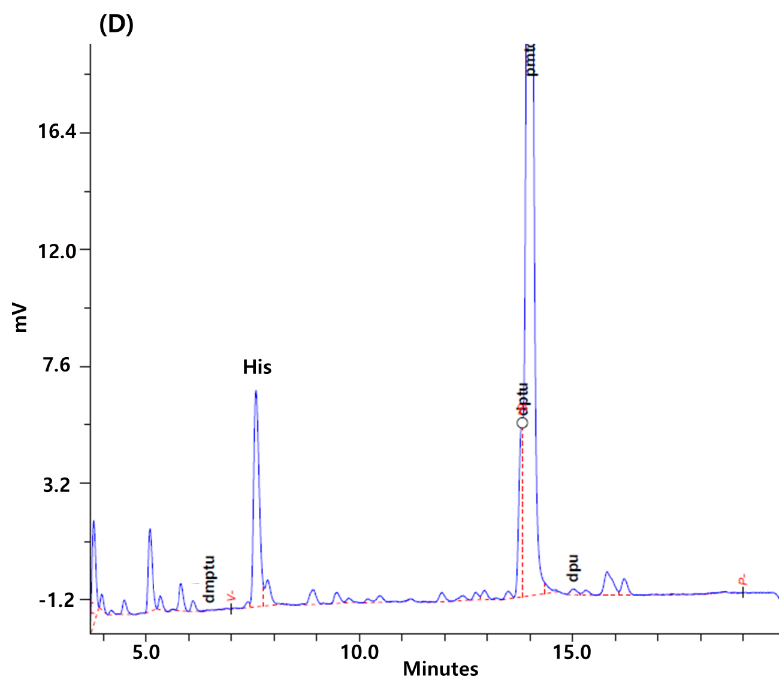

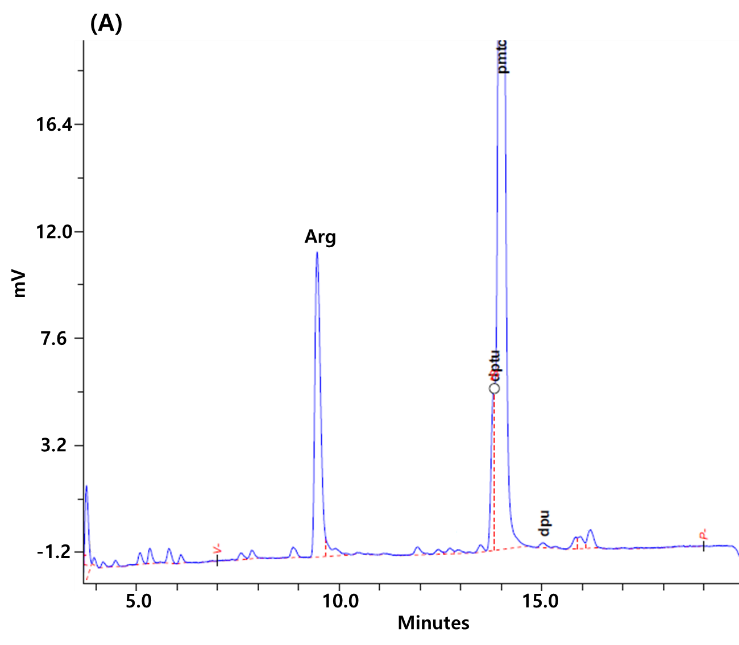

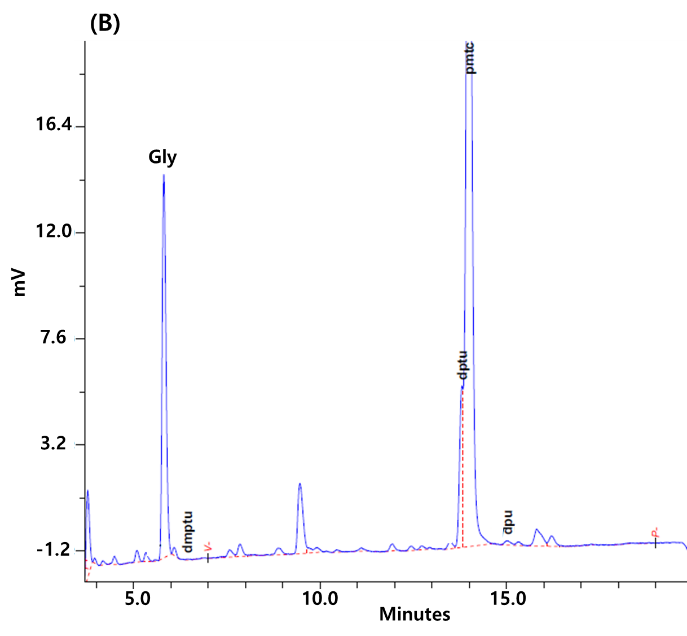


**Supplementary Figure S4.** Edman sequencing results for the second (**A**), the third (**B**), the fourth (**C**), and the fifth residue (**D**) of the purified Z domain expressed with OpgY. The amino acid sequence (TAG-Z domain) is shown in **Table S2**.


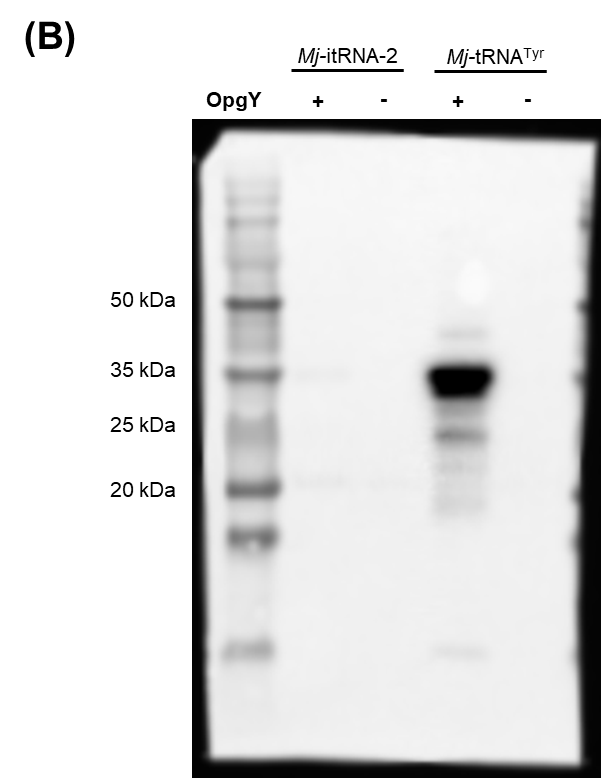

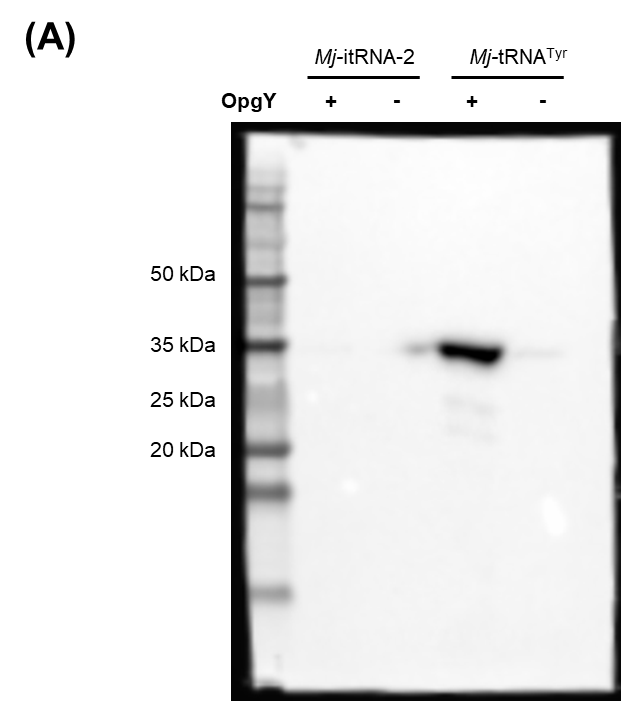


**Supplementary Figure S5.** **(A)** The unprocessed western blot data figure 4 panel A, **(B)** The unprocessed western blot data figure 4 panel B.

Table S1. Primers used in this study

| Primer | DNA sequence (5′→3′) |
| --- | --- |
| Primer 1 | AACCTTCATATGGTGTCAGAATCACTACGTATTATTTTTG |
| Primer 2 | CCGAACTGCAAGCTGATGTTA |
| Primer 3 | TAACATCAGCTTGCAGTTCGG |
| Primer 4 | GATTACTGCGGAAGATACCAG |
| Primer 5 | CTGGTATCTTCCGCAGTAATC |
| Primer 6 | AAGGTTCTGCAGTCAGACCAGACGGTTGCCCGGAACAAAC |
| Primer 7 | AACCTTCATATGCGGTGACGAAGCGTAATAAACTGTAG |
| Primer 8 | GAGCAGGAAGCTGCGGAGCTCAAGCG |
| Primer 9 | CGCTTGAGCTCCGCAGCTTCCTGCTC |
| Primer 10 | GTAAAGGTTCTTCGCTGCATCAAGGCTTCCAG |
| Primer 11 | CTGGAAGCCTTGATGCAGCGAAGAACCTTTAC |
| Primer 12 | GGACGCTATCCTGCTGCATGCGGAAGTTCTGG |
| Primer 13 | CCAGAACTTCCGCATGCAGCAGGATAGCGTCC |
| Primer 14 | AAGGTTCTGCAGTTAAGCAATGGTACGTTGGATCTCG |
| Primer 15 | TATGATACCATGGAAAGGATCCAAAAAGCTTATAGCGGCCGCAAAACTCGAGCACCACCACCACCACCACA |
| Primer 16 | TCGATGTGGTGGTGGTGGTGGTGCTCGAGTTTTGCGGCCGCTATAAGCTTTTTGGATCCTTTCCATGGTATCA |
| Primer 17 | AACCTTGAATTCAAAAGATCTTTTAAGAAGGAGATATATAGAGTAAAGGAGAAGAACTTTTCACTGGAG |
| Primer 18 | CTCAGCTAATTAAGCTTTTTGTAGAGCTC |
| Primer 19 | AATTCCGAATTCGTAGACAACAAATTCAACAAAGAAC |
| Primer 20 | TTCCTTCTCGAGTCATTAGTGATGGTGGTGGTGATGCCAC |
| Primer 21 | GATCCTAGGGTTCTGGCGGTTCAGGTG |
| Primer 22 | AATTCACCTGAACCGCCAGAACCCTAG |

Table S2. Amino acid sequences of the proteins used in this study

| Protein | Amino acid sequence |
| --- | --- |
| TAG-Z domain  (pSPEL542) | *RGSHHHHHHGSMAVDNKFNKEQQNAFYEILHLPNLNEEQRNAFIQSLKDDPSQSANLLAEAKKLNDAQAPW |
| TAG-11.3.3  (pSPEL530) | *SKGEELFTGVVPILVELDGDVNGHKFSVRGEGEGDATYGKITLKLICTTGKIPVPWPTLVTTCGYGVQCFARYPDHMKRHDFFKSAMPEGYVQERTISFKDDGKFKTRAEVKFEGDTIVNRIKLKGIDFKEDGNILGHKLEYNFNSHDVYIMADKQKTGIKANFKIRHNVEDGSVQLADHYQQNTPIGDGPVRLPDNHYLLTQSVISKDPNEKRDHMVLHEFVTAAGITHGIDELYK |
| GST-TAG-Z domain  (pSPEL236) | MSPILGYWKIKGLVQPTRLLLEYLEEKYEEHLYERDEGDKWRNKKFELGLEFPNLPYYIDGDVKLTQSMAIIRYIADKHNMLGGCPKERAEISMLEGAVLDIRYGVSRIAYSKDFETLKVDFLSKLPEMLKMFEDRLCHKTYLNGDHVTHPDFMLYDALDVVLYMDPMCLDAFPKLVCFKKRIEAIPQIDKYLKSSKYIAWPLQGWQATFGGGDHPPKSDLVPRGS*GSGGSGEFVDNKFNKEQQNAFYEILHLPNLNEEQRNAFIQSLKDDPSQSANLLAEAKKLNDAQAPWHHHHHH |
| MTF | VSESLRIIFAGTPDFAARHLDALLSSGHNVVGVFTQPDRPAGRGKKLMPSPVKVLAEEKGLPVFQPVSLRPQENQQLVAELQADVMVVVAYGLILPKAVLEMPRLGCINVHGSLLPRWRGAAPIQRSLWAGDAETGVTIMQMDVGLDTGDMLYKLSCPITAEDTSGTLYDKLAELGPQGLITTLKQLADGTAKPEVQDETLVTYAEKLSKEEARIDWSLSAAQLERCIRAFNPWPMSWLEIEGQPVKVWKASVIDTATNAAPGTILEANKQGIQVATGDGILNLLSLQPAGKKAMSAQDLLNSRREWFVPGNRLV |
| IF-2 | MTDVTIKTLAAERQTSVERLVQQFADAGIRKSADDSVSAQEKQTLIDHLNQKNSGPDKLTLQRKTRSTLNIPGTGGKSKSVQIEVRKKRTFVKRDPQEAERLAAEEQAQREAEEQARREAEESAKREAQQKAEREAAEQAKREAAEQAKREAAEKDKVSNQQDDMTKNAQAEKARREQEAAELKRKAEEEARRKLEEEARRVAEEARRMAEENKWTDNAEPTEDSSDYHVTTSQHARQAEDESDREVEGGRGRGRNAKAARPKKGNKHAESKADREEARAAVRGGKGGKRKGSSLQQGFQKPAQAVNRDVVIGETITVGELANKMAVKGSQVIKAMMKLGAMATINQVIDQETAQLVAEEMGHKVILRRENELEEAVMSDRDTGAAAEPRAPVVTIMGHVDHGKTSLLDYIRSTKVASGEAGGITQHIGAYHVETENGMITFLDTPGHAAFTSMRARGAQATDIVVLVVAADDGVMPQTIEAIQHAKAAQVPVVVAVNKIDKPEADPDRVKNELSQYGILPEEWGGESQFVHVSAKAGTGIDELLDAILLQAEVLELKAVRKGMASGAVIESFLDKGRGPVATVLVREGTLHKGDIVLCGFEYGRVRAMRNELGQEVLEAGPSIPVEILGLSGVPAAGDEVTVVRDEKKAREVALYRQGKFREVKLARQQKSKLENMFANMTEGEVHEVNIVLKADVQGSVEAISDSLLKLSTDEVKVKIIGSGVGGITETDATLAAASNAILVGFNVRADASARKVIEAESLDLRYYSVIYNLIDEVKAAMSGMLSPELKQQIIGLAEVRDVFKSPKFGAIAGCMVTEGVVKRHNPIRVLRDNVVIYEGELESLRRFKDDVNEVRNGMECGIGVKNYNDVRTGDVIEVFEIIEIQRTIA |

Asterisks (*) indicate the stop codon position for the incorporation of unnatural amino acids.

Table S3. tRNA genes used in this study

| tRNA | DNA sequence (5′→3′) |
| --- | --- |
| itRNA-1 | AACCTT**GTGCAC**GCAAAAGAGGCAGCGGCTAACTAAGCGGCCTGCTGACTTTCTCGCCGATCAAAAGGCATTTTGCTATTAAGGGATTGACGAGGGCGTATCTGCGCAGTAAGATGCGCCCCGCATTCGCGCGGTAGTTCAGCAGGGCAGAACGGCGGGCTCTAAACCCGCATGGCGCTGGTTCAAATCCGGCCCGCGCAACCAAATTCGAAAAGCCTGCTCAACGAGCAGGCTTTTTTGCATG**CTCGAG**AACCTT |
| itRNA-2 | AACCTT**GTGCAC**GCAAAAGAGGCAGCGGCTAACTAAGCGGCCTGCTGACTTTCTCGCCGATCAAAAGGCATTTTGCTATTAAGGGATTGACGAGGGCGTATCTGCGCAGTAAGATGCGCCCCGCATTCGCGCGGTAGTTCAGCAGGGCAGAACGGCGGGCTCTAAACCCGCATGGCGCTGGTTCAAATCCGGCCCGCGCAACCAAATTCGAAAAGCCTGCTCAACGAGCAGGCTTTTTTGCATG**CTCGAG**AACCTT |

The tRNA sequence is underlined and placed between the ProK promoter and the ProK terminator.

The restriction enzyme cleavage site is indicated in bold.
